# Impact of COVID-19 on care-home mortality and life expectancy in Scotland

**DOI:** 10.1093/ageing/afab080

**Published:** 2021-04-29

**Authors:** Jennifer K Burton, Martin Reid, Ciara Gribben, David Caldwell, David N Clark, Peter Hanlon, Terence J Quinn, Colin Fischbacher, Peter Knight, Bruce Guthrie, David A McAllister

**Affiliations:** Institute of Cardiovascular and Medical Sciences, University of Glasgow, Glasgow G31 2ER, UK; Public Health Scotland, Meridian Court, Glasgow G2 6QE, UK; Public Health Scotland, Gyle Square , Edinburgh EH12 9EB, UK; Public Health Scotland, Gyle Square , Edinburgh EH12 9EB, UK; Public Health Scotland, Gyle Square , Edinburgh EH12 9EB, UK; Institute of Health and Wellbeing, University of Glasgow, Glasgow G12 8RZ, UK; Institute of Cardiovascular and Medical Sciences, University of Glasgow, Glasgow G31 2ER, UK; Public Health Scotland, Gyle Square , Edinburgh EH12 9EB, UK; Public Health Scotland, Gyle Square , Edinburgh EH12 9EB, UK; Advanced Care Research Centre, University of Edinburgh, Old Medical School, Edinburgh EG8 9AG, UK; Public Health Scotland, Meridian Court, Glasgow G2 6QE, UK; Institute of Health and Wellbeing, University of Glasgow, Glasgow G12 8RZ, UK

**Keywords:** care home, mortality, life expectancy, COVID-19, vaccination, older people

## Abstract

**Background:**

COVID-19 deaths are commoner among care-home residents, but the mortality burden has not been quantified.

**Methods:**

Care-home residency was identified via a national primary care registration database linked to mortality data. Life expectancy was estimated using Makeham–Gompertz models to (i) describe yearly life expectancy from November 2015 to October 2020 (ii) compare life expectancy (during 2016–18) between care-home residents and the wider population and (iii) apply care-home life expectancy estimates to COVID-19 death counts to estimate years of life lost (YLL).

**Results:**

Among care-home residents, life expectancy in 2015/16 to 2019/20 ranged from 2.7 to 2.3 years for women and 2.3 to 1.8 years for men. Age–sex-specific life expectancy in 2016–18 in care-home residents was lower than in the Scottish population (10 and 2.5 years in those aged 70 and 90, respectively). Applying care home-specific life expectancies to COVID-19 deaths yield mean YLLs for care-home residents of 2.6 and 2.2 for women and men, respectively. In total YLL care-home residents have lost 3,560 years in women and 2,046 years in men. Approximately half of deaths and a quarter of YLL attributed to COVID-19 were accounted for by the 5% of over-70s who were care-home residents.

**Conclusion:**

COVID-19 infection has led to the loss of substantial years of life in care-home residents aged 70 years and over in Scotland. Prioritising the 5% of older adults who are care-home residents for vaccination is justified not only in terms of total deaths, but also in terms of YLL.

## Key Points

- Deaths among care-home residents account for a considerable proportion of all mortality in older adults.
- Life expectancy in care-home residents during the pandemic fell by almost 6 months.
- Over 5,600 years of life were lost (YLL) by care-home residents in Scotland who died with COVID-19.
- During the COVID-19 pandemic a smaller proportion of deaths among care-home residents occurred in hospitals.
- Prioritising care-home residents for vaccination is justified in terms of total deaths and YLL.

## Background

COVID-19 has severely impacted people living in care homes [1, 2]. Residents have experienced significant morbidity and mortality [3, 4] and also reduced access to routine healthcare and other services and disconnection from family and friends [5]. Striking the correct balance between minimising loss of life from COVID-19 and maintaining the quality of life of care-home residents has proved controversial [6, 7].

Care homes in Scotland are home to ~5% of the population aged over 70 years [8, 9]. Compared with older people in the general population, people living in care homes in the United Kingdom are known to have higher mortality [10–12]. Care homes largely care for people approaching the end of their lives [13], so higher mortality is expected, but there is limited contemporary population-level data on the life expectancy of care-home residents [14]. Moreover, while the life expectancy (i.e. the notional years of life lost [YLL]) of people dying with COVID-19 in the community has been estimated [15, 16], there are no published data on the YLL among care-home residents who died with COVID-19.

Such information is difficult to apply to individuals (who may live for a considerably longer or shorter duration than their life expectancy), but it is crucial for designing vaccination and other preventive strategies that minimise the total YLL across the population. Prior to the (imminent) availability of effective vaccines, COVID-19 care-home policies involved trading-off short-term restrictions to quality of life against the prevention of COVID-19 morbidity and mortality. However, these trade-offs will be different in someone near the end of life compared with someone with a longer life expectancy. Indeed, this trade-off applies not only to COVID-19, but also to many drug therapies aimed at extending life rather than treating symptoms. For all these purposes, decision-making by care-home residents, their families and policymakers, requires information on the life expectancy of care-home residents.

Due to high-quality routinely collected data on care-home residency status, it is possible in Scotland to address this need. Using these data, for care-home residents and other older people, we describe 5-year trends in age- and sex-specific mortality, trends in life expectancy among care-home residents and estimate the mortality impact of COVID-19 including YLL up to November 2020.

## Methods

### Design, population and data sources

A population-wide dynamic cohort study was created using the Community Health Index (CHI) register, based on the CHI number (a unique identifier variable used across the National Health Services [NHS] in Scotland). All adults aged 70 years and over, registered with a general practitioner (GP) in Scotland were included. Residency was categorised into care home or not, based on the presence of the institution flag on records of those living at care-home addresses, applied at time of GP registration or address change. Data for care-home residents were linked to the National Records of Scotland (NRS) mortality data, which record all deaths certified in Scotland in any location. In addition, for 2020, cases of COVID-19 where an individual died were collected using data from the REACT-SCOT population-wide case–control study [17].

### Exposures

A care home in Scotland is defined as a facility providing 24-hour care to its residents with no regulatory distinction between ‘residential’ and ‘nursing’ homes [18]. While some care homes provide care for younger adults (e.g. people with learning disability) [19], we restricted this analysis to the older adult population aged 70 years and over.

Care-home residency was defined as the presence of a relevant Institution Code on the CHI register, which was available from 2007. Individuals were defined as care-home residents if they had a first CHI-flag indicating care-home residency on or after 8 years prior to each month. Eight years was chosen so that the ‘look-back’ period for earliest month fell after the recording of care home had been established within the CHI database. This analysis was focused on long-stay residents in care homes. This method does not capture temporary stays for respite and intermediate care (where CHI registered address does not change) or those who die before a change of address is recorded within primary care.

We examined the accuracy of the CHI Institution flag, against a new measure of care-home status, which was created in 2020 in response to the COVID-19 pandemic and is thus only available for 2020. A detailed description of the care-home status measure is provided in the Supplementary Material A1. Briefly, the Unique Property Reference Number (UPRN), an Ordnance Survey product that uniquely identifies each property in the United Kingdom, was assigned to all addresses in the CHI register for all individuals (>5.5 million) alive at any time during 2020. Each UPRN was then mapped to a definitive list of addresses of registered care-home services held by the Care Inspectorate [9]. Using the control arm of the REACT case–control study (which is an age, sex and general practice area stratified random sample of all individuals in Scotland), we then calculated the sensitivity and specificity of the CHI care-home flag as a measure of care-home residency status on the 1 August 2020 against the new care-home status measure.

### Outcomes

Mortality was examined for all care-home residents using NRS mortality data. Causes of death were grouped based on the underlying cause of death derived from the death certificate into the following categories: respiratory (including pneumonia) [ICD1 J00–J99 and COVID-19—U07], dementia [F01, F03, G30], circulatory [I00–I97], cancer [C00–C97] and other. Deaths in hospital were identified from the location of death recorded on the death certificate.

Life expectancy estimates for the general population were obtained from published NRS national life tables [20] held on the Human Mortality Database [21].

For the YLL calculations for death with COVID-19, we included all deaths within 28 days of a positive SARS-CoV-2 polymerase chain reaction test result and/or with COVID-19 recorded as a cause of their death (underlying or otherwise). Counts of deaths with COVID-19 by age, sex and care-home residency for all individuals aged 70 years and over in Scotland were obtained from the REACT case–control study.

### Covariates

Individuals were categorised by age, sex among care-home residents and the general Scottish population. For those who died, age at death and year of death were calculated. Age at care-home admission was calculated based on date of birth and the date that the institution code was first applied to the CHI register. This was to allow inclusion of those who move between care-home services but remain resident in a care-home over several years.

### Statistical analysis

The statistical analysis was conducted in two stages. First for care-home residents individual-level data were aggregated. For each calendar month from October 2015 to November 2020, we summed events and person-time by sex among care-home residents aged 70 or older at the time of death. We summed person-time and deaths overall, by cause and where the place of death was a hospital. Person-time was calculated similarly as the number of days in the month minus any days prior to an individual being admitted to a care home and any days after the individual had died. We also obtained aggregated data on deaths for all individuals aged 70 years or older in Scotland up to September 2020. Events and person-time were aggregated (i) by sex and calendar month for all years, (ii) by age, sex and each 12 months running from November 2015 to October 2020 and (iii) by age, sex and calendar year from 2016 to 2018 inclusive. The former period was chosen as these were the latest data available for care homes; the latter period was chosen to allow comparison with published life tables from the National Records for Scotland.

Secondly, using the MortalityLaws package in R (version 2.0, R Core team, Austria), Makeham–Gompertz models were fitted to the age- and sex-specific mortality rates to obtain age- and sex-specific life expectancy estimates for each year and for the 3-year period from 2016–18. Uncertainty was propagated to the estimates by fitting Makeham–Gompertz models for 1,000 samples from Binomial distributions independently sampled from each stratum (defined by the age, sex, time period and care-home residency status) where the probability parameter was the number of events divided by the population at risk at the onset of each time period to obtain a distribution of estimates and summarised via the 2.5th and 97.5th percentiles. For the 2016–18 period, life expectancy was also calculated non-parametrically using life tables, and both analyses were compared with the NRS-calculated life expectancy (which are also based on life tables) for the entire Scottish population.

The care-home resident and NRS age–sex-specific life expectancy estimates were applied to the age–sex distribution of individuals who died with COVID-19 (from the REACT case–control study) to calculate the YLL among people aged 70 years and over. The YLL due to COVID-19 ignoring care-home residency (i.e. based solely on the NRS population life tables) and accounting for care-home residency (based on the life expectancy estimates described above) were then calculated as the age–sex-specific remaining life expectancy for each individual who had died.

### Data governance

Standard Public Health Scotland (PHS) policies were followed in conducting analyses using data held within PHS for the purposes of monitoring population health and supporting policymaking.

## Results

In 2017, 26,056 women and 11,086 men aged 70 years and over were resident in care homes, representing 6 and 4% of the whole population in this age group. Of these 25% had resided in a care home for <12 months with a further 25% for <24 months with a median stay of 1.85 years for men and 2.12 years for women (Supplementary Figure S1 for full distribution).

### Validation of CHI flag

The sensitivity and specificity of the CHI care-home flag compared with the UPRN care-home flag in 2020 was excellent (96%), with only minor variation across the NHS Board regions.

### Trends in mortality and life expectancy

Deaths among care-home residents in all years 2015/16–19/20 accounted for a considerable proportion of all mortality in older adults, ~19% for men and 30% for women. Differences between 2020 and previous years were seen in April and May. In the month of April during the years 2016–19, 29–32% of deaths in women were among care-home residents, whereas in April 2020 the equivalent figure was 42%. Differences of similar magnitude were seen in May among women and in April and May among men. In 2019/20 13% of female and 18% of male care-home resident deaths took place in hospital, lower than the 24–25% and 16–19% in 2015/16–18/19 (Table 1).

**Table 1:** Care-home resident deaths

Sex	November to October	% of all deaths of those aged 70 years and older that were deaths of care-home residents	% of care-home residents’ deaths that occurred in hospital
Men	2015/16	3,470/18,227 (19%)	865/3,470 (25%)
	2016/17	3,355/19,068 (18%)	820/3,355 (24%)
	2017/18	3,575/19,841 (18%)	849/3,575 (24%)
	2018/19	3,525/19,127 (18%)	833/3,525 (24%)
	2019/20	4,389/19,618 (22%)	779/4,389 (18%)
Women	2015/16	6,857/22,534 (30%)	1,324/6,857 (19%)
	2016/17	6,748/23,415 (29%)	1,180/6,748 (17%)
	2017/18	7,137/24,089 (30%)	1,171/7,137 (16%)
	2018/19	6,555/22,757 (29%)	1,050/6,555 (16%)
	2019/20	7,970/23,364 (34%)	1,024/7,970 (13%)

For this table the period was October to September (rather than November to October that was used for the formal analyses). This was done to allow comparison with quarterly deaths data released by NRS for people aged 70 and older.

Supplementary Figure S2 shows mortality in care-home residents compared with the general Scottish population. As well as the large peak in spring 2020, there was a peak in winter 2017/18 (which is known to have been a severe year for seasonal influenza) [22] and a smaller peak in winter 2019/20.

**Figure 1:**
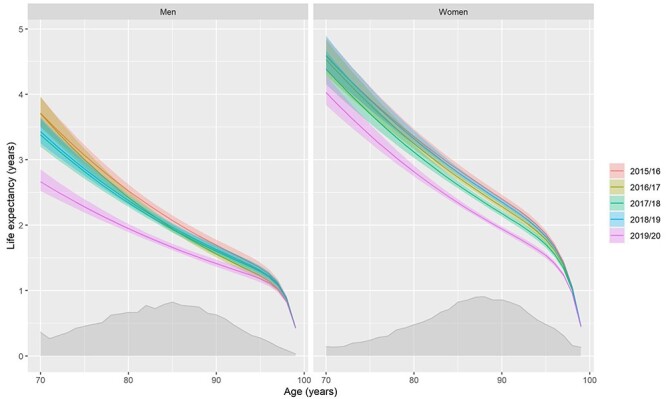
Age- and sex-specific life expectancy estimates for care-home residents with uncertainty intervals, 2015/16–19/20. Shaded area denotes the age and sex distribution of the care-home population in 2017 (to retain clarity in the figure and since absolute values of probability densities are not easily interpretable without further calculation; no secondary axis is shown).

This increased mortality in November 2019 to October 2020, and to a lesser extent the same period in 2017/18, is reflected in life expectancy estimates for these periods. Among women aged 70 years and over living in care-homes life expectancy was 2.7, 2.6, 2.5, 2.7 and 2.3 years, respectively, for 2015/16–19/20. For men, corresponding figures were 2.3, 2.1, 2.1, 2.1 and 1.8 years. Compared with the highest year, life expectancy in care-home residents fell by ~2–2.5 months in 2017/18 and ~5.5 months in 2019/20 (Table 2, Figure 1).

**Table 2:** Life expectancy estimates for November to October 2015/16–19/20 for care-home residents

Sex	Life expectancy (years)	2015/16	2016/17	2017/18	2018/19	2019/20
Women	Absolute	2.74 (2.69–2.81)	2.63 (2.58–2.70)	2.52 (2.47–2.58)	2.70 (2.65–2.76)	2.27 (2.23–2.33)
Men	Absolute	2.27 (2.21–2.35)	2.16 (2.11–2.24)	2.11 (2.06–2.19)	2.14 (2.09–2.21)	1.78 (1.73–1.84)
Women	Reduction from 2015/16	–	–	0.21 (0.13–0.30)	0.04 (0.05–0.12)	0.47 (0.39–0.55)
Men	Reduction from 2015/16	–	–	0.16 (0.06–0.25)	0.13 (0.03–0.23)	0.49 (0.41–0.58)

**Figure 2:**
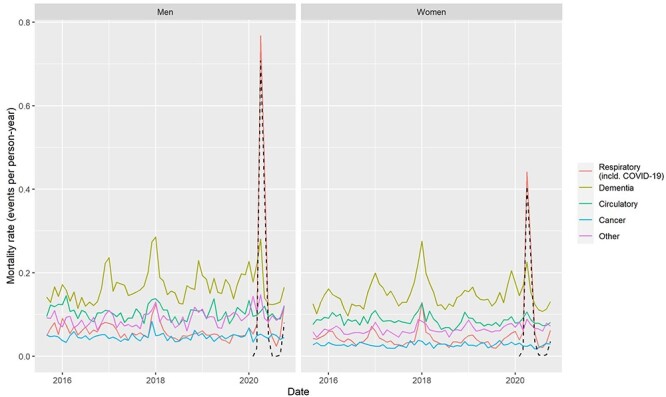
Sex-specific mortality by cause in care-home residents, October 2015 to November 2020. Dotted line indicates deaths with COVID-19 as the underlying cause that are a subset of respiratory deaths. All other causes are mutually exclusive.

Figure 2 shows the mortality by underlying cause of death for the same period. In 2019/20 more than half of the mortality peak was deaths attributed to respiratory disease, mostly COVID-19, with an increase also seen in circulatory diseases. Dementia was the second largest single recorded cause of care-home deaths in 2019/20.

### Life expectancy and YLL in people dying with COVID-19

**Figure 3:**
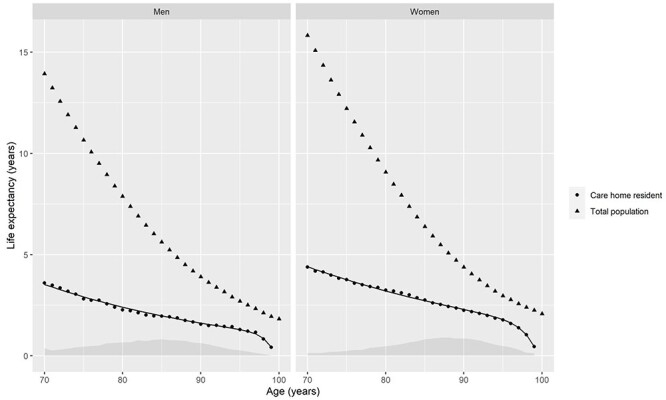
Age- and sex-specific life expectancy in care-home residents compared with the age- and sex-specific general Scottish population, 2016–18. Shaded area denotes the age and sex distribution of the care-home population in 2017 (to retain clarity in the figure and since absolute values of probability densities are not easily interpretable without further calculation; no secondary axis is shown). Calendar-year data were used here rather than 12 months, which maximised the months spent within the pandemic in order to allow comparison between the care-home residents life expectancy estimates and that obtained from the NRS life tables.

Figure 3 shows the 2016–18 age- and sex-specific life expectancy for adults aged 70 years and over. It shows both the standard total population estimates (from NRS) and estimates we produced for care-home residents in this analysis. Care-home residents have substantially shorter life expectancies than those living in non-institutional settings, with larger differences at younger ages (e.g. ~10 years aged 70 and 2.5 years aged 90). Averaging over the age distribution of care-home residents alive in 2017 led to a difference of around 5 years.

Table 3 shows the estimated YLL in people aged 70 years and over obtained by applying these age–sex and residency-specific life expectancy estimates to the 1,370 women and 943 men who were care-home residents, and the 927 women and 1,204 men who were not care-home residents, who died of COVID-19. Among care-home residents, the impact of estimating YLL using care home-specific life expectancy estimates rather than general population life tables was large, leading to a difference of ~4 years; on using care home-specific life expectancy estimates for women, the estimated average YLL fell from 6.24 years per death to 2.60 years per death. For men, the equivalent figures were 6.73 and 2.17.

**Table 3:** YLL among over-70s due to COVID-19 March to November 2020

	Women		Men	
Life expectancy method used for YLL calculation	NRS solely	NRS and care home	NRS solely	NRS and care home
Care-home deaths	1,370	1,370	943	943
Not care-home deaths	927	927	1,204	1,204
Overall deaths	2,297	2,297	2,147	2,147
Total YLL care home	8,553	3,560	6,349	2046
Total YLL not care home	7,652	7,652	9,520	9,520
**Total YLL overall**	**16,204**	**11,211**	**15,870**	**11,566**
Average YLL care home	6.24	2.60	6.73	2.17
Average YLL not care home	8.25	8.25	7.91	7.91
**Average YLL overall**	**7.05**	**4.88**	**7.39**	**5.39**

The total YLL is the sum of all YLL of those who died, and the average YLL is this number divided by the total number of deaths. ‘NRS solely’ indicates that national life tables were used to estimate the YLL for all deaths regardless of care-home residency. ‘NRS and care home’ indicates that care home-specific life expectancy estimates were used to estimate deaths for care-home residents, and national life tables were used for deaths in individuals not resident in care homes. The NRS life tables are based on all deaths, including care-home residents and so will slightly underestimate life expectancy (LE) in individuals who are not residents of care-home residents.

Care-home residents only account for ~5% of the Scottish population of people aged 70 years and older, but they account for 52% of all COVID-19 deaths in this age group (and 44% of all COVID-19 deaths). Using care home-specific life expectancy estimates to calculate YLL in care-home residents, the average YLL for care-home residents remained >2 years for both men and women, corresponding to a total YLL of 3,560 years in women and 2,046 years in men. At a whole population level, recalculating YLL based on care home-specific life expectancy has a large effect on the overall YLL attributable to COVID-19. In the over-70s this fell from 7.05 to 4.88 years in women and 7.39 to 5.39 years in men.

## Discussion

This analysis provides the first population-level data reporting care-home life expectancy and evaluating the impact of COVID-19 on those living in care homes in terms of YLL in the UK NHS context, and we make several important observations.

Life expectancy in care-home residents during the pandemic fell by almost 6 months. The estimated YLL due to COVID-19 is substantial for care-home residents at around two and half years per death for women and 2 years per death for men, corresponding to a total of ~3,600 YLL in women and 2,000 YLL in men. Although care-home residents are only ~5% of the population aged 70 years and older, care-home resident YLL represents around a quarter of all YLL among those in that age group who died with COVID-19.

In the period prior to the pandemic life expectancy in care-home residents was substantially lower than in non-care-home residents; consequently the YLL per death is estimated to be ~4 years lower when care home-specific life expectancy rather than national life tables are used for the calculation. Since around half of COVID-19 deaths in those aged 70 years and over were among care-home residents, this has a large impact at the level of the population, with the average YLL being around 5 years rather than seven among all those aged 70 years and over. Thus, calculations of YLL for COVID-19 should use care home-specific life expectancy estimates rather than standard national life tables.

Our findings are broadly consistent with studies reporting COVID-19 mortality among care-home residents in Scotland [3], elsewhere in the UK [4, 23], and internationally [24, 25]. In one UK analysis based on data (including primary care data) from Wales, there was found to be excess mortality among care-home residents even after accounting for frailty [23]. When we expressed the mortality impact in terms of life expectancy, the impact of the pandemic on mortality from December 2019 to November 2020 was more than 2-fold larger than that seen during 2017/18, which included a severe influenza season. However, this observation needs to be seen in the context of the considerable efforts to prevent entry of SARS-CoV2 to care homes, and the fact that only 32% of care homes had any cases of COVID-19 [26].

Some may be surprised that the life expectancy in care-home residents was not shorter than the 2–2.5 years we observed. It is important to note that our analysis does not include individuals who died before a change of address was recorded in primary care and as such is applicable only to residents who survive beyond this initial period of days to weeks before an updated prescription is required. Nonetheless, our findings are consistent with our previous work on a smaller sample of ~22,000 long-stay (≥6 weeks) care-home resident deaths in Scotland using the Scottish Care-Home Census data over 4 years [19] and with a previous study in England and Wales, which found that the 1-year mortality was 26% [10].

Our findings also have implications for studies examining the impact of COVID-19 and the pandemic response on wider society, where these rely on estimates of YLL or related metrics such as quality adjusted life years (QALYs) and disability adjusted life years (DALYs). We and others have reported YLL with COVID-19 [15, 27] of ~10 years in the general population. We accounted for multimorbidity [15] but not care-home residency. Our current findings suggest that this is an important omission; the combination of a large difference in life expectancy and a large proportion of COVID-19 deaths occurring in care-homes means that estimates that do not account for care-home residency substantially overestimate the total YLL in the population.

### Limitations of the study

This analysis is based on Scotland-wide registration systems and is therefore highly representative. Use of anonymised, linked routinely collected data is inclusive and not reliant on individual consent—an important consideration when analysing data on the population living in care homes as it will lead to reduced bias [28]. GP registration is necessary as a means of accessing primary healthcare in Scotland and thus is likely to represent a highly representative sampling frame when the focus is on individuals aged 70 years and over. Our validation work using UPRN in 2020 is reassuring in terms of the accuracy of the CHI-based identification of care-home status. Nonetheless, under-ascertainment of care-home residents is possible, though difficult to quantify. Data are published on care-home places (~40,000 in 2020), but occupancy is not routinely collected or reported and some care homes operate exclusively for non-resident short stays and respite care. Previous analyses identified ~35,000 long-stay residents in Scotland’s care homes at all ages [19], and our data broadly align with these figures. However, we are likely to have missed those with very short care-home stays (e.g. those discharged to care homes for end of lifecare who die before their registered address is changed), and our findings cannot be applied to such individuals.

It is important to note that the life expectancy estimates presented are the average across care-home residents and for individual residents the age at which death occurs will vary considerably. The data presented are reported by age and sex but not adjusted for any other clinical conditions. This may not be a major issue for co-morbidity, which is less predictive of mortality risk among those living in care homes [10], but information on dependency or frailty [29], which is not systematically recorded in routine healthcare data, would likely have led to more precise estimates of life expectancy and may have caused us to overestimate the YLL in those dying with COVID-19.

Some misclassification of cause of deaths is also likely to have occurred. While this does not affect the life expectancy estimates, as these are based on all-cause mortality, it may have affected the YLL calculation since this was based on cause-specific mortality. One alternative to examining cause-specific deaths is to calculate the excess deaths during the pandemic compared with the previous 5 years. Excess death calculations include both direct and indirect deaths and so have a different interpretation to the deaths we present here. Moreover, excess death calculations are typically based on 5 years of historic data (unlike the 4-year historic comparison presented here). Nonetheless, it is notable that comparing recent deaths to the previous 4-year period (Table 1) there are ~2,329 excess deaths, a similar figure to the 2,313 cause-specific deaths we used to estimate YLL, suggesting bias due to misclassification would be minimal.

### Implications for practice, policy and future research

The most immediate priority we advocate is prioritising care-home residents and staff for vaccination. Our rationale is that by targeting 5% of the older adult population we can prevent around half of deaths and a quarter of YLL. Assuming vaccines are similarly efficacious across groups, vaccination strategies that specifically target care homes are likely to have a larger impact on YLL than those that do not. Furthermore, these benefits may extend to those providing care to this vulnerable group as social care staff have experienced excess mortality during the pandemic [30].

Even if vaccines rapidly bring the pandemic to an end, the observed 2-year life expectancy means that care-home residents will have had severely restricted access to family and friends for around half of their remaining lives. Balancing reducing COVID mortality risk by restricting visiting and activity outside the care home with harm from social isolation has been one of the most difficult policy decisions during the pandemic. While COVID-19 has proved a measurable outcome, the mental health impacts and well-being of residents have been more difficult to quantify objectively. Future pandemic planning therefore needs to engage with residents and their families to discover how best this balance could be struck. In communal settings, which are both people’s homes and places of work, where many residents have cognitive impairment and where there are concerns about financial and even criminal consequences of these decisions, this process will be challenging and likely contentious. Nonetheless, accurate estimates of life expectancy will help inform such debates.

Moving forwards the mortality impacts have resulted in greater governmental awareness of the needs of care homes in practical aspects such as procurement of and access to personal protective equipment [1, 31]. More complete understanding of applied questions, such as usual mortality and life expectancy, requires dynamic, comprehensive national care-home data inclusive of short stays and temporary placements, collated by care homes themselves with identifiers to facilitate linkage to other data sources [32]. Improving the visibility of care-home residents within routine data systems can facilitate more informed and inclusive analyses to support the delivery of routine care and help inform modelling and planning for future pandemics and other significant events.

While not the main focus of this study, it is interesting to note the small trend before COVID-19 away from deaths in hospital [13]. This likely reflects existing policy to encourage planned death in ‘homely’ settings rather than hospitals, but it is unclear whether the larger drop in deaths in hospital during COVID-19 was appropriate or harmful and merits further exploration.

Beyond COVID-19, providing life expectancy estimates and historical comparisons for the care-home population is valuable. Individual and societal planning for care needs in older age relies on understanding the likely need for and costs of care to ensure needs are properly met. Understanding life expectancy may help clinicians, residents and their families make decisions about their healthcare, facilitating more informed discussions around their priorities and wishes.

## Conclusions

COVID-19 infection has led to the loss of substantial years of life in care-home residents aged 70 years and over in Scotland. Mortality impacts have been disproportionate on a vulnerable group. Life expectancy among those living in care homes is consistently shorter than those of the same age and sex living elsewhere. This emphasises the need to prioritise the care of this group and consider how best to support their preferences and values and maximise their quality of life.

## Supplementary Material

aa-21-0096-File002_afab080

## Data Availability

All aggregated data (and analysis code) is available at https://github.com/ChronicDiseaseEpi/care_homes_covid_le. For access to the individual level anonymised data, apply to the Electronic Data Research and Innovation Service (eDRIS), information available at www.isdscotland.org/Products-and-Services/eDRIS/
