# Supplementary material for: Impact of COVID-19 on care-home mortality and life expectancy in Scotland
Source: Age Ageing. 2021 Apr 29;50(4):1029–37. doi: 10.1093/ageing/afab080 (PMC8135527; doi:10.1093/ageing/afab080)
Supplement: aa-21-0096-File002_afab080 [file aa-21-0096-file002_afab080.docx]

Impact of COVID-19 on Care-Home Mortality and Life Expectancy in Scotland

# SUPPLEMENTARY DATA

# Appendix – UPRN seeding of CHI

Public Health Scotland undertook work to seed the Community Health Index (CHI) database, of all General Practice registrations in Scotland, with a Unique Property Reference Number (UPRN), based on the individual’s registered address. This work was done in collaboration with the Improvement Service, utilising their Data Hub service. UPRN was derived from the Ordnance Survey Address Base

Address fields (3 address lines plus postcode) were extracted from the PHS CHI monthly download (dated 03-Aug-2020) from current records of persons still alive in Scotland or who died since 1st January 2020. This yielded 3,208,951 unique address strings relating to 5,828,951 people. Some minimal formatting and pre-processing was carried out in order to extract a Town entity from the address lines, trim leading spaces, format postcodes, and apply a series of rules to expand abbreviations at end of address lines (e.g. “RD” to “ROAD”, “DR” to “DRIVE” etc.). Files of unique address strings were processed at DataHub - https://datahub.scot/home/ - using the Non-residential UPRN seeding templates and programs. These returned 5 categories of output file with best matching UPRN attached to the input strings where applicable. The categories of output were EXCELLENT/GOOD/FAIR/MULTIPLE/NO_MATCH and the output is available from: <https://www.scadr.ac.uk/sites/default/files/CURLreport2311%20-%20A%20guide%20to%20CHI-UPRN%20Residential%20Linkage.pdf>

Additional manual work was undertaken to identify care-home addresses from the unmatched sample (441,550 unique addresses). Addresses were matched based on care-home name and partial postcode to known care-home addresses by a single reviewer, experienced in care-home address matching. Additional review was undertaken of addresses which had matched manually to identify care-homes in which there were no recorded residents or a mismatch of >10 registered places vs records in CHI. This work was done independently from the Institution Flag allocation. This manual work was necessary to maximise correct allocation of care-home addresses to UPRNs identifying care-homes.

# Supplementary figure 1 – cumulative length of care home stay


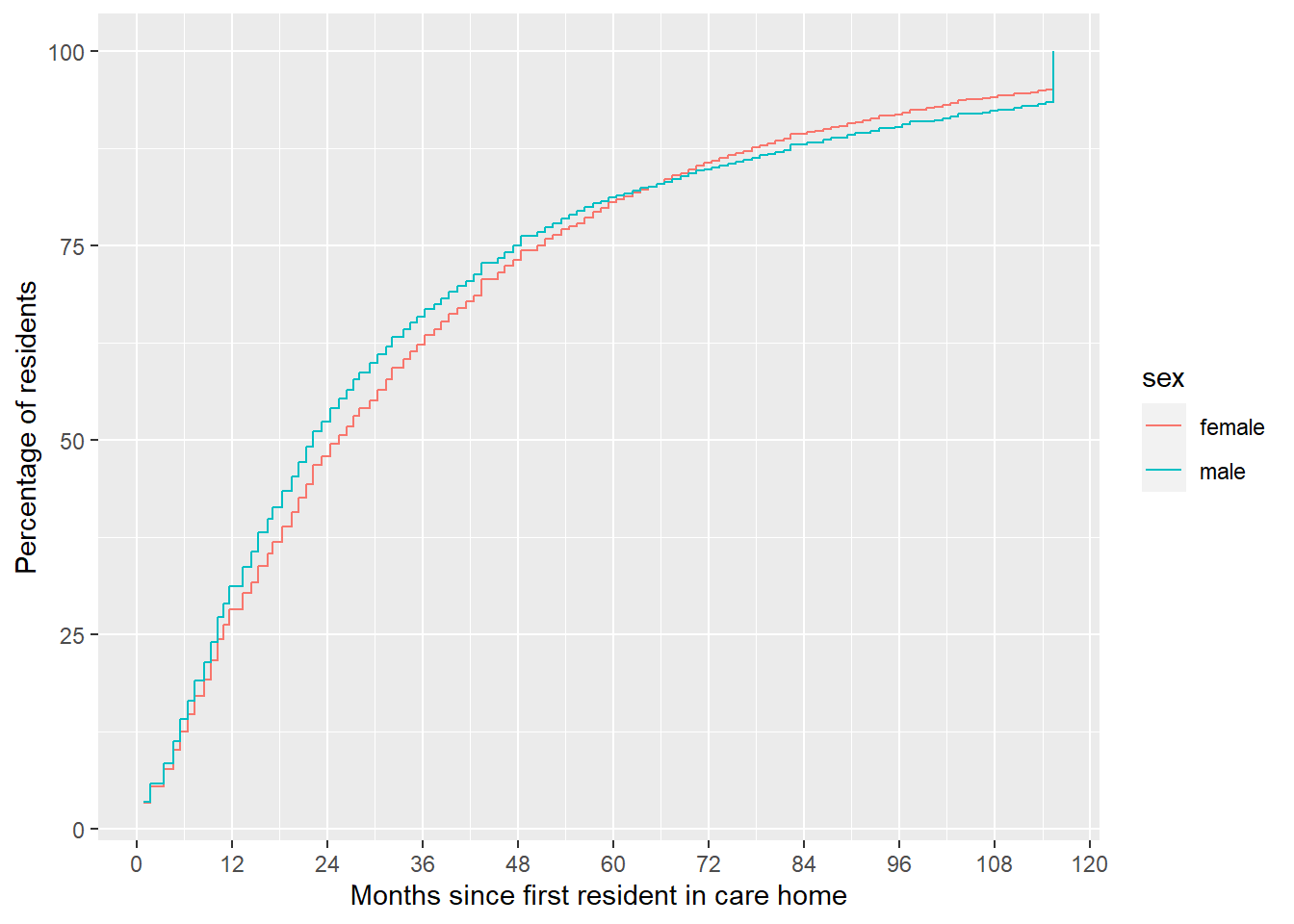


*Red lines are rates in women and blue in men.*

# Supplementary figure 2: Sex-specific mortality in care-home residents compared to the general Scottish population mortality, October 2015 to November 2020


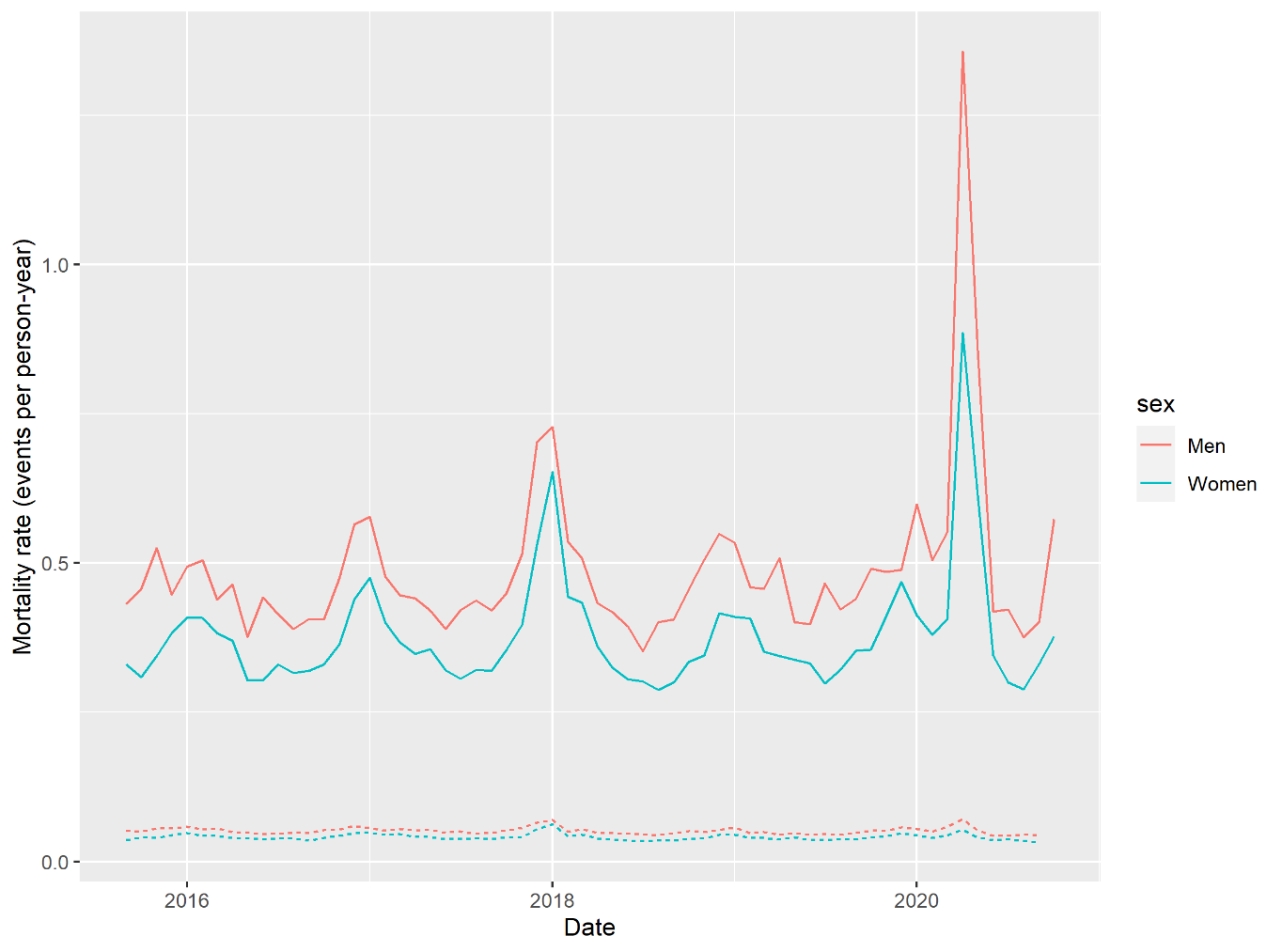


Red lines are rates in women and blue in men. The rate is per (single) person year. Solid lines indicate care-home residents aged 70 years and over and dotted lines the general population aged 70 years and over comparison.

# References

1. Gordon AL, Goodman C, Achterberg W, Barker RO, Burns E, Hanratty B, et al. Commentary: COVID in care homes-challenges and dilemmas in healthcare delivery. Age and Ageing. 2020 Aug 24;49(5):701-5.

2. Salcher-Konrad M, Jhass A, Naci H, Tan M, El-Tawil Y, Comas-Herrera A. COVID-19 related mortality and spread of disease in long-term care: first findings from a living systematic review of emerging evidence. medRxiv. 2020:2020.06.09.20125237.

3. Burton JK, Bayne G, Evans C, Garbe F, Gorman D, Honhold N, et al. Evolution and effects of COVID-19 outbreaks in care homes: a population analysis in 189 care homes in one geographical region of the UK. The Lancet Healthy Longevity. 2020;1(1):e21-e31.

4. Morciano M, Stokes J, Kontopantelis E, Hall I, Turner AJ. Excess mortality for care home residents during the first 23 weeks of the COVID-19 pandemic in England: a national cohort study. medRxiv. 2020:2020.11.11.20229815.

5. Comas-Herrera A, Salcher-Konrad M, Baumbusch J, Farina N, Goodman C, Lorenz-Dant K, et al. Rapid review of the evidence on impacts of visiting policies in care homes during the COVID-19 pandemic LTCcovidorg International Long-Term Care Policy Network [serial on the Internet]. 2020 [cited 2020 4th November]: Available from: <https://ltccovid.org/wp-content/uploads/2020/11/Rapid-review-of-evidence-on-impacts-of-visiting-policies-in-care-homes-during-the-COVID-pandemic-LSE068110.pdf>.

6. Andersen L, Anobile A, Bolton P, Brown S, Catlin R, Caslake B, et al. Open letter: Infection prevention and control should never be at the expense of compassionate care. 2020 [cited 2020 24th November]; Available from: <https://www.nursingtimes.net/opinion/open-letter-infection-prevention-and-control-should-never-be-at-the-expense-of-compassionate-care-16-10-2020/>.

7. Bell D, Comas-Herrera A, Henderson D, Jones S, Lemmon E, Moro M, et al. COVID-19 mortality and long-term care: a UK comparison. International Long-Term Care Policy Network, [serial on the Internet]. 2020 [cited 2020 15th October]; August 2020.: Available from: <https://ltccovid.org/wp-content/uploads/2020/08/COVID-19-mortality-in-long-term-care-final-Sat-29-v1.pdf>.

8. National Records of Scotland. NRS 2019 Mid-Year Population Estimates by NHS Board Area. 2020; Available from: <https://www.nrscotland.gov.uk/statistics-and-data/statistics/statistics-by-theme/population/population-estimates/mid-year-population-estimates/mid-2019>.

9. Care Inspectorate. Datastore. 2020; Available from: <https://www.careinspectorate.com/index.php/publications-statistics/93-public/datastore>.

10. Shah SM, Carey IM, Harris T, DeWilde S, Cook DG. Mortality in older care home residents in England and Wales. Age and Ageing. 2013;42(2):209-15.

11. Kinley J, Hockley J, Stone L, Dewey M, Hansford P, Stewart R, et al. The provision of care for residents dying in U.K. nursing care homes. Age and Ageing. 2014 May;43(3):375-9.

12. McCann M, O'Reilly D, Cardwell C. A Census-based longitudinal study of variations in survival amongst residents of nursing and residential homes in Northern Ireland. Age and Ageing. 2009 Nov;38(6):711-7.

13. Finucane AM, Bone AE, Evans CJ, Gomes B, Meade R, Higginson IJ, et al. The impact of population ageing on end-of-life care in Scotland: projections of place of death and recommendations for future service provision. BMC Palliative Care. 2019 2019/12/12;18(1):112.

14. Williams ES, Dinsdale H, Eayres D, Tahzib F. Impact of nursing home deaths on life expectancy calculations in small areas. Journal of Epidemiology and Community Health. 2004;58(11):958-62.

15. Hanlon P, Chadwick F, Shah A, Wood R, Minton J, McCartney G, et al. COVID-19 - exploring the implications of long-term condition type and extent of multimorbidity on years of life lost: a modelling study [version 1; peer review: 1 approved]. Wellcome Open Research. 2020;5(75).

16. Oh IH, Ock M, Jang SY, Go DS, Kim YE, Jung YS, et al. Years of Life Lost Attributable to COVID-19 in High-incidence Countries. J Korean Med Sci. 2020;35(32):e300-e.

17. McKeigue PM, Weir A, Bishop J, McGurnaghan SJ, Kennedy S, McAllister D, et al. Rapid Epidemiological Analysis of Comorbidities and Treatments as risk factors for COVID-19 in Scotland (REACT-SCOT): A population-based case-control study. PLOS Medicine. 2020;17(10):e1003374.

18. Care Inspectorate. Care homes for adults. 2015 [cited 2019 18th January]; Available from: <http://www.careinspectorate.com/index.php/care-homes-for-adults>.

19. Burton JK, Lynch E, Love S, Rintoul J, Starr JM, Shenkin SD. Who lives in Scotland's care homes? Descriptive analysis using routinely collected social care data 2012-16. The journal of the Royal College of Physicians of Edinburgh. 2019;49(1):12-22.

20. National Records of Scotland. Life Expectancy in Scotland, 2017-2019. 2020 [updated 24 September 2020; cited 2020 October]; Available from: <https://www.nrscotland.gov.uk/statistics-and-data/statistics/statistics-by-theme/life-expectancy/life-expectancy-in-scotland/2017-2019>.

21. Shkolnikov V, Barbieri M, Wilmoth J. The Human Mortality Database. 2020; Available from: <https://www.mortality.org/>.

22. Vestergaard LS, Nielsen J, Krause TG, Espenhain L, Tersago K, Bustos Sierra N, et al. Excess all-cause and influenza-attributable mortality in Europe, December 2016 to February 2017. Eurosurveillance. 2017;22(14):30506.

23. Hollinghurst J, Lyons J, Fry R, Akbari A, Gravenor M, Watkins A, et al. The impact of COVID-19 on adjusted mortality risk in care homes for older adults in Wales, UK: a retrospective population-based cohort study for mortality in 2016–2020. Age and Ageing. 2020.

24. Fisman DN, Bogoch I, Lapointe-Shaw L, McCready J, Tuite AR. Risk Factors Associated With Mortality Among Residents With Coronavirus Disease 2019 (COVID-19) in Long-term Care Facilities in Ontario, Canada. JAMA Network Open. 2020;3(7):e2015957-e.

25. Comas-Herrera A, Zalakain J, Lemmon E, Henderson D, Litwin C, Hsu A, et al. Mortality associated with COVID-19 in care homes: international evidence. LTCcovidorg International Long-Term Care Policy Network [serial on the Internet]. 2020 [cited 2020 3rd November ]; 14 October: Available from: <https://ltccovid.org/wp-content/uploads/2020/10/Mortality-associated-with-COVID-among-people-living-in-care-homes-14-October-2020-4.pdf>.

26. Public Health Scotland. Discharges from NHSScotland Hospitals to Care Homes between 1 March and 31 May 2020. 2020: Available from: <https://beta.isdscotland.org/find-publications-and-data/population-health/covid-19/discharges-from-nhsscotland-hospitals-to-care-homes/>.

27. Wetzler HP, Wetzler EA, Cobb HW. COVID-19: How Many Years of Life Lost? medRxiv. 2020:2020.06.08.20050559.

28. Goodman C, Baron N, Machen I, Stevenson E, Evans C, Davies S, et al. Culture, consent, costs and care homes: Enabling older people with dementia to participate in research. Aging & Mental Health. 2011;15(4):475-81.

29. Andrew MK, McElhaney JE. Age and frailty in COVID-19 vaccine development. The Lancet. 2020:S0140-6736(20)32481-8.

30. Hodgson K, Grimm F, Vestesson E, Brine R, Deeny S. Briefing: Adult social care and COVID-19. The Health Foundation; 2020 [cited 2020 25th October]; Available from: <https://www.health.org.uk/publications/reports/adult-social-care-and-covid-19-assessing-the-impact-on-social-care-users-and-staff-in-england-so-far>.

31. House of Commons Public Accounts Committee. COVID-19: Government procurement and supply of Personal Protective Equipment2021 [cited 2021 1st March]: Available from: <https://committees.parliament.uk/publications/4607/documents/46709/default/>.

32. Burton J, Goodman C, Guthrie B, Gordon A, Hanratty B, Quinn T. Closing the UK care home data gap - methodological challenges and solutions. International Journal of Population Data Science. 2020;5(4):doi: 10.23889/ijpds.v5i4.1391.
